# 

**Published:** 1892-02

**Authors:** 


					﻿
Entereda tthe P^tri Oftre at Austin. Tfj'<u.a»See'>nd-C'd<i> Mai M^&
VOL. VII.
v FEimr a in',1 mp».
No. 8.
DANIELS
MEDICAL
JOURNAL
 SnbsCTi^foh;, JarJbp a'Yeart in Adyanice^—r^ihglc^opjes, Tweply-fiveCents.
F,: E. DANIEL., M.D^ Editor and Publisher,‘AUSTIN, TEXAS.
Steam Book and Jor Printer, Austin, Texas
				

